# *Copaifera* of the Neotropics: A Review of the Phytochemistry and Pharmacology

**DOI:** 10.3390/ijms19051511

**Published:** 2018-05-18

**Authors:** Rafaela da Trindade, Joyce Kelly da Silva, William N. Setzer

**Affiliations:** 1 Programa de Pós-Graduação em Biotecnologia, Universidade Federal do Pará, 66075-900 Belém, Brazil; rcabral@ufpa.br (R.d.T.); joycekellys@ufpa.br (J.K.d.S.); 2 Programa de Pós-Graduação em Química, Universidade Federal do Pará, 66075-900 Belém, Brazil; 3 Department of Chemistry, University of Alabama in Huntsville, Huntsville, AL 35899, USA; 4 Aromatic Plant Research Center, 615 St. George Square Court, Suite 300, Winston-Salem, NC 27103, USA

**Keywords:** copaiba, oleoresin, essential oil, sesquiterpenoids, diterpenoids, biological activity, molecular targets

## Abstract

The oleoresin of *Copaifera* trees has been widely used as a traditional medicine in Neotropical regions for thousands of years and remains a popular treatment for a variety of ailments. The copaiba resins are generally composed of a volatile oil made up largely of sesquiterpene hydrocarbons, such as β-caryophyllene, α-copaene, β-elemene, α-humulene, and germacrene D. In addition, the oleoresin is also made up of several biologically active diterpene acids, including copalic acid, kaurenoic acid, alepterolic acid, and polyalthic acid. This review presents a summary of the ecology and distribution of *Copaifera* species, the traditional uses, the biological activities, and the phytochemistry of copaiba oleoresins. In addition, several biomolecular targets relevant to the bioactivities have been implicated by molecular docking methods.

## 1. Introduction to the Genus *Copaifera*

The copaiba trees belong to the genus *Copaifera*, family Fabaceae, and subfamily Caesalpinoideae. The genus was described the first time by Marcgraf and Piso in 1638, who employed the name “Copaiba” without designating the species [1]. In 1760, Nicolaus Joseph Von Jacquin described the species *Copaiva officinalis* in the work *Enumeratio Systematica Plantarum* [2]. Afterwards, in the year 1764, Carl von Linnaeus did a more detailed study of the genus in the work *Species Plantarum*, in which he described the type species *Copaifera officinalis* (Jacq.) L. [3]. There are more than 70 *Copaifera* species distributed throughout the world, with widespread occurrence in Central and South America; there are also four species found in Africa and one species found on the island of Borneo, situated in the Pacific Ocean [4]. Brazil is the country with the greatest biodiversity of *Copaifera* with 26 species and 8 varieties [5].

The vernacular name copaíba probably originated from the Tupi-Guarani and alludes to the names used by indigenous peoples, copaíva and copahu (kupa’iwa and kupa’u, respectively), which refers to the tree exudate, in reference to the oil stored in its interior [6]. Sixteenth-century records produced by chroniclers during the Brazilian colonization report the widespread use of copaiba oil among the natives as anti-inflammatory and healing agents, and also for esoteric purposes, such as aphrodisiac and contraceptive [4,6,7]. This natural product is known and valued to the present day, mainly in the Amazon region, where the rural population has little access to industrialized pharmaceutical products and public health care [6,8].

The copaiba trees have shrub or arboreal habits, can reach up to 40 m height and 4 m diameter at breast height (dbh), have slow growth, and can live up to 400 years [6]. Their cylindrical trunks contain intercellular secretory channels arranged in bands of marginal axial parenchyma, the lumen from secretory cells is formed schizogenously, and the oleoresin is synthesized in parenchyma cells of the canal. The species have alternate leaves, which are pinnate with 2–12 pairs of leaflets (opposite, alternate, or subopposite), usually glabrous, and may have translucent points and glands at the base of the marginal vein; they have small and interpetiolar stipules and are generally deciduous. The inflorescences are alternate panicles and the flower buds are protected by small bracts; they have small flowers, numerous and sessile, which are monoclamids with a tetramer chalice that forms short tubes and contains internally hirsute sepals. The androecium holds 10 free stamens, glabrous fillets, and oblong and rimose anther; and the gynoecium presents a sessile ovary with two elongate ovules, filiform style, and globular and papillary stigma. The fruits are bivalved, dehiscent, laterally compressed, and monospermic. The seed is a pendulum, oblong-globose, covered by abundant white or yellow aril, and lacking endosperm [1,9,10,11].

Although the *Copaifera* genus has been extensively studied taxonomically, there are still difficulties in identifying some species, mainly due to their intricate floral morphology and absence of reproductive structures in the samples studied. With regard to the Amazonian species, the scarcity of field information and illustrations of specimens comprise the main limitations for botanical descriptions of the group. These taxonomic problems have restricted the advance of chemical and pharmacological research, limited the industrial and rational uses of resin oils and wood, and have also hampered the development of projects, plans for sustainable management, and conservation of commercially targeted species [9,12].

The main economic contributions of *Copaifera* species have been wood and oleoresins. Among *Copaifera* species that are used in the production of oleoresins, *C. reticulata* is the most frequent, representing 70% of the production [6]. Copaiba oleoresin is one of the most important renewable natural remedies for the indigenous people from the Amazon region and its use is widely diffused due its various pharmacological properties [13]. The oleoresin is a transparent, colored liquid with variable viscosity, and is constituted by a nonvolatile fraction composed of diterpenes and a volatile fraction composed of sesquiterpenes [14,15]. Its chemical profile may vary according to species, seasonal and climatic characteristics of the environment, soil type and composition, and rainfall index. Biotic pressures, such as insect predation and pathogen infection, also cause differences in oleoresin composition [16,17]. The extraction of copaiba oil is done through the perforation of the trunk with a punch, and the resin is collected with the help of a polyvinyl chloride (PVC) pipe, through which the oil flows and is then stored. This practice is mainly done by plant extraction; therefore, the product of several trees is often mixed, resulting in an additional obstacle to the botanical identity of the copaiba trees. In addition, the lack of parameters to characterize the oil and to perform quality control of the botanical drug also constitutes an obstacle for the registration and exportation of herbal products containing copaiba [18,19].

## 2. Ecology and Distribution of *Copaifera*

The genus *Copaifera* is native to tropical regions of Latin America, an area of great species diversity [1]. Distributed widely in the Americas, stretching from Mexico to northern Argentina, the genus also occurs in West Africa and Asia [20]. The greatest richness of species occurs in Brazil, where they are distributed from the north to the south of the country. The most common species are *C. multijuga* Hayne, which is found in the Amazonas, Pará and Rondônia states; *C. reticulata* Ducke that occurs in Amapá, Pará and Roraima;, and *C. langsdorffii* Desf., which can occur from the northern to southern regions of Brazil [5]. Other species have more restricted distribution, such as *C. guyanensis* Desf. (Amazonas), *C. majorina* Dwyer (Bahia), *C. cearensis* Huber ex Ducke (Ceará, Bahia, Piauí and Rio de Janeiro), *C. elliptica* Mart. (Goias and Mato Grosso), *C. paupera* (Herzog) Dwyer (Acre), and *C. lucens* Dwyer (Bahia, Espírito Santo, Minas Gerais, Rio de Janeiro, São Paulo) [5]. Although many species of *Copaifera* have wide occurrence within the Brazilian territory, and may occur in different phytogeographic domains (e.g., *C. langsdorffii*), some feature endemism, such as *C. trapezifolia* Hayne, which occurs in an extremely disturbed region of the Atlantic rainforest, of which only 11.6% of the natural vegetation cover remains [21]. Thus, morphological, physiological, and ecological studies are highly relevant for the preservation of species and their natural environment [11]. A study conducted in the Minas Gerais state on the geographical distribution and environmental characteristics of arboreal species showed that *C. langsdorffii* has wide occurrence throughout the whole state, where latosol type soil predominates, but additionally has a preference for ustic soils (62%) [22].

In relation to the ecological group, copaíba are classified as long-living, late secondary, and climax tree species, demanding of light but tolerant to shade [23]. They are considered generalists because they are adapted to a wide variety of environments. They can occur in floodplains, riparian forest, and streams of the Amazon basin and the forests of the Cerrado in the center of Brazil [24]. *C. langsdorffii*, for example, has great ecological plasticity, occurring in several biomes, such as Cerrado, Atlantic Forest, Caatinga, and Amazon rainforest [23]. *Copaifera* species have great plasticity in relation to edaphic conditions; they occur in areas with fertile soil and well-drained soil and in areas with very poor acidic soils, such as Cerrado fields. They grow well on sandy and clayey soils and generally occupy the forest canopy [25,26].

Phenological studies on *Copaifera* are important for the rational use of the species and for the preparation of management plans [27]. The reproduction of copaibas occurs from the fifth year of growth after planting in a climax forest ecosystem [6]. *C. multijuga*, commonly found in the Amazon, blooms in the rainy season—between the months of December–April—and fructifies between April and July [27]. Blooming of *C. reticulata* occurs from January to March, with fruiting from March to August, lasting into October [26,28]. *C. langsdorffii*, observed in the Tijuca Forest, Rio de Janeiro, blooms between March and April and fructifies between August and September. Another survey carried out near Campinas, São Paulo state, showed that flowering of *C. langsdorffii* occurs in the middle of the rainy season (December–February), with development of fruit during the dry season (April–September) [29,30]. The phenophases of *C. officinalis* were monitored in the municipality of Boa Vista (Roraima state, Brazil), and showed that the flowering of the species occurs between the months of September and November and the fruiting from November to March. Depending on the stage of fruit ripening, the dehiscence can begin in January, in which the seeds enveloped by the aril are exposed, allowing for their dispersal [28]. 

*Copaifera* is a hermaphrodite plant of mixed reproduction with a predominance of allogamy. The trees are generally bee pollinated (melittophily), and *Apis mellifera* and *Trigona* spp. are its main pollinating agents [25]. *C. langsdorffii* has high fecundity, producing large quantity of fruits in a short period of time. Its seeds have low nutritional value, mainly composed of carbohydrates, but can attract a wide variety of animals with a general diet [30]. The dispersion of the copaiba seeds occurs mainly in zoocoric and barocoric forms [27,28]. Some vertebrates, such as birds and mammals, have been observed visiting the fruits of *Copaifera* [31]. Its seeds have morphological characteristics that fit the ornithocoria syndrome, mainly because they are black with colored, fleshy arils, which, after being swallowed, can be regurgitated intact and remain viable for germination [32]. A study revealed that 10 species of Passeriformes, such as *Ramphastos toco*, *Cyanocorax cristatellus*, and *Turdus rufiventris*, visited the fruits of *C. langsdorffii*. Likewise, monkeys of the species *Eriodes arachnoides* and *Cebus paella* also eat the fruits of *C. langsdorffii* [31]. Copaiba seeds may also present hydrocoric dispersion due to their frequent occurrence near waterways [25]. Copaiba seeds are of conventional behavior and may be conserved in the long term ex situ, with dormancy due to the deposition of coumarin in the tegument, and its germination is of the epigene type [25]. A tree can produce from 2 to 3 kg of seeds [33].

The population density of copaiba trees in an area is usually very low. It is possible to find only one tree every 5 ha, but they may occur in densities of one to two trees per hectare. The production of oleoresin by species is fairly variable and can be influenced by genetic differences among species, habitat, soil, and intensity of exploitation [34]. The production of oleoresin per tree ranges from 100 mL to 60 L per year. In addition, not all trees produce oil [24]. Therefore, detailed investigations regarding extraction methods and equipment that do not harm the plant, correlation of genetic data to botanical identification of species, floristic inventory of copaiba populations, and ecological studies on its ecosystems are indispensable for the sustainable and rational use of this resource [35,36].

## 3. Traditional Uses of *Copaifera*

### 3.1. Medicinal Uses

In Pará state (Amazon region, Brazil), people of all ages and social classes consider copaiba one of the most important natural remedies from the Amazon region. Several parts and preparations of the plant are used in folk medicine [24]. The oleoresin or bark decoction is used as an anti-inflammatory and contraceptive by native people from the Brazilian Amazon. The topical application of oil on the skin serves to heal wounds. It is used in massages on the head to cure paralysis, pains, and convulsions. In Amapá state, it is recommended to soak a cotton ball in oil and place on tumors, ulcers, or hives. The daily intake of two drops of oil mixed with one tablespoon of honey is indicated for inflammation, syphilis, bronchitis, and cough [6,37,38]. In Venezuela, the oil is used to prepare a patch that is applied to heal ulcers and wounds, and the decoction of the bark in the form of a bath is used to combat rheumatism, to wash infected wounds such as dog bites, and to use as an anti-tetanus [37,38]. A tea from the seeds is also used as a purgative and for treatment of asthma. In northern Brazil, the practice of “embrocation” (applying oil directly to the throat) is common to treat throat infections [39]. In Belém, the “garrafada”—an infusion of the bark sold in bottles—is currently used as a substitute for the oleoresin due to the difficulty in obtaining the oil in the city [38]. 

Copaiba has a wide range of ethnopharmacological indications, including for the treatment of: cystitis, urinary incontinence, gonorrhea, and syphilis; respiratory ailments, including bronchitis, strep throat, hemoptysis, pneumonia, and sinusitis; infections in the skin and mucosa, such as dermatitis, eczema, psoriasis, and wounds; ulcers and lesions of the uterus; leishmaniasis and leucorrhea; anemia; headaches; and snake bites. It is also used for its aphrodisiac, stimulant, anti-inflammatory, antiseptic, anti-tetanus, antirheumatic, antiherpetic, anthelminthic, anticancer, antitumor (prostate tumors), and antiparalytic properties [4,6,26,38,40]. *Copaifera* species are used by people of Igarapé Miri (Pará state) for healing wounds [41].

Studies have shown that the ingestion of high doses of copaiba oil can cause adverse side effects, such as gastrointestinal irritation, sialorrhea, and central nervous system depression. A dose of 10 g may cause symptoms of intolerance, nausea, vomiting, colic and diarrhea, and exanthema. Prolonged use may cause kidney damage and topical reactions in susceptible individuals [39,42]. Thus, the advance in pharmacological and quality control studies of copaiba formulations sold at herbal markets is indispensable for the safe use of this plant drug. 

### 3.2. Human Nutrition

Copaiba oil was approved in the United States as a food additive and is used in small amounts as a flavoring agent in foods and beverages [43].

### 3.3. Cosmetic Uses

The species of *Copaifera* are intensively pursued for inclusion in the cosmetics market due to their therapeutic properties and fragrant value of their oils [44]. Copaiba oil is currently used in the cosmetic industry as a fixative for perfumes and perfuming soaps [38]. As an emollient, bactericidal, and anti-inflammatory agent, copaiba oil is used in the production of soaps, lotions, creams and moisturizers, bath foams, shampoos, and hair conditioners [6,24]. In addition, it aids in the treatment of dandruff and acne [38,45]. Despite its fragrant value, little information regarding its odorant potential is available in the literature [44].

### 3.4. Fuel

As a renewable source of hydrocarbons, the use of copaiba oil as an ecologically clean fuel has been evaluated. Experimental plantations were started in the early 1980s near Manaus, Brazil to test its viability as an alternative energy source to fossil fuels [7]. For potential use as fuel, a combination with diesel oil in a ratio of 9:1 (diesel oil to copaiba) has been recommended [6]. Various reports indicate that the liquid can be poured directly into the fuel tank of a diesel-powered car and the vehicle will run normally, with a bluish exhaust smoke being the only noticeable difference [46]. Traditionally, the oil is used in lamps as fuel for lighting [24].

### 3.5. Wood

The copaiba trees are considered hardwoods with high demand due to their properties of strength, as well as insect and xylophagous fungi repellency. The wood is saturated with oil and resin and has been used in both shipbuilding and civil construction, especially in the manufacture of steam caves, pool cues, and decorative and furniture coverings. It is also used in the preparation of lumbers, rafters, door and window frames, and boards in general, including for agricultural implements, general carpentry, flooring furniture, coatings, lamination, plywood sheets. The wood has a high content of lignin and is very good for the production of alcohol and charcoal. *C. langsdorffii* has traditionally been exploited extensively for charcoal in the Cariri Region, south of Ceará [24,47]. 

### 3.6. Veterinary Uses

In southern Pará state, farmers have used copaiba oil to prevent foot-and-mouth infection in cattle. The oil is poured on the floor next to the salt lick so that when cattle approach to eat salt, they step in oil soaking their feet [24]. When wounded, some animals lick and rub their bodies in the oil that flows from the trees [24].

### 3.7. Other Uses

Hunters often hunt under the copaiba tree during fruiting because the seeds and oil attract animals [24]. The oleoresin is used in the photographic industry to improve image clarity in areas of low contrast and resolution. The resin has also been used in paper making, as an additive for butadiene in the production of synthetic rubber, as a source of a chiral substrate in the synthesis of biomarkers of sediment and oil residues, and as fixative in the manufacture of varnish, perfume, and paints used in the painting of porcelain, fabrics, and for dying cotton yarn [6,24,38].

## 4. Essential Oil Chemistry of *Copaifera*

The major components of the essential oils from *Copaifera* species are summarized in Table 1. In general, copaiba oils derived from *Copaifera* oleoresins are rich in sesquiterpene hydrocarbons and often dominated by β-caryophyllene [15]. Some copaiba oils, however, also show significant concentrations of diterpene acids, which are generally analyzed as their methyl esters [15]. A perusal of internet sources of copaiba oil suggests that the most important commercial sources of copaiba oil are *C. langsdorffii*, *C. officinalis*, and *C. reticulata*, and the most prized copaiba oils are rich in β-caryophyllene. The oleoresin essential oils from these three *Copaifera* species can have as much as 33% (*C. langsdorffii*), 87% (*C. officinalis*), and 68% (*C. reticulata*) β-caryophyllene (see Table 1).

## 5. Nonvolatile Chemistry of *Copaifera*

**Figure 1:**
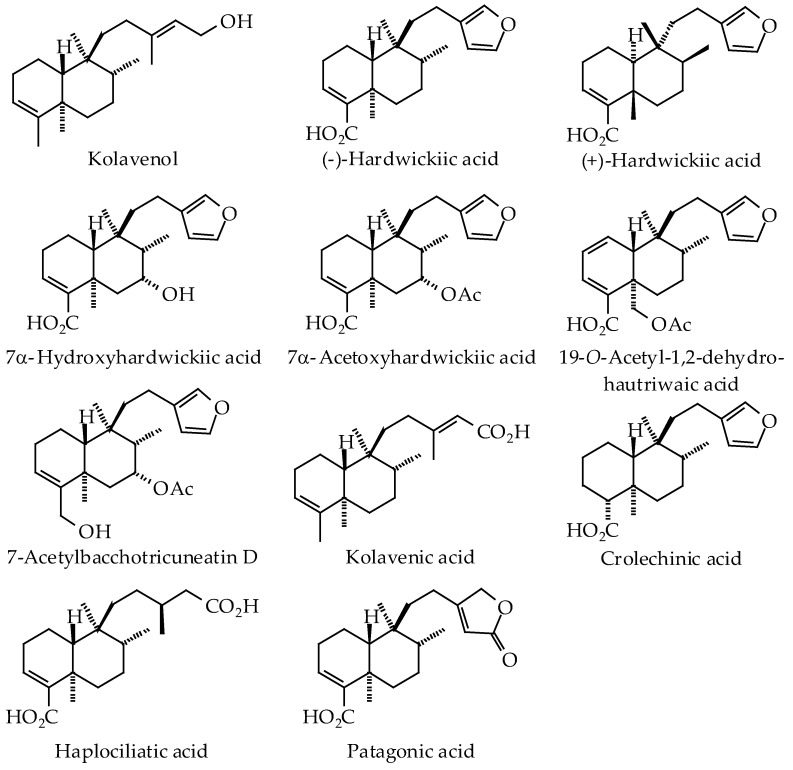
Clerodane diterpenoids found in *Copaifera* species.

**Figure 2:**
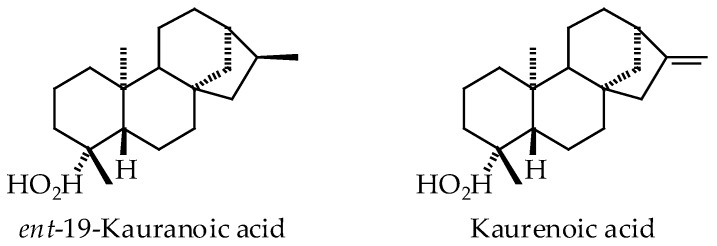
Kaurane diterpenoids found in *Copaifera* species.

**Figure 3:**
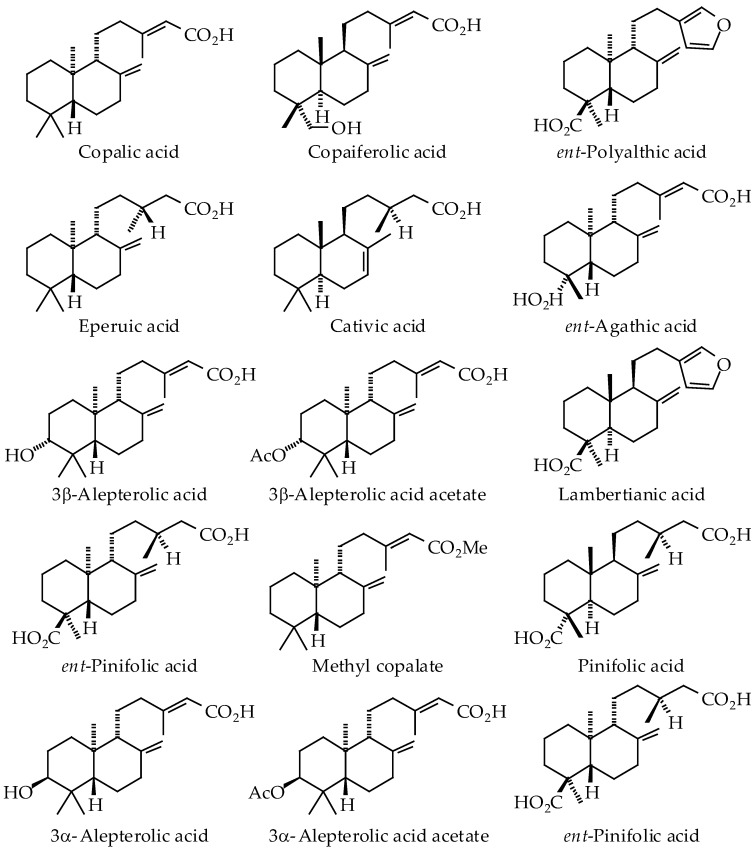
Labdane diterpenoids found in *Copaifera* species.

The oleoresins of several *Copaifera* species have been shown to be rich sources of clerodane, kaurane, and labdane triterpenoids (Figure 1, Figure 2 and Figure 3, Table 2). In particular, *C. langsdorffii* resin is composed of biologically active copalic acid [70,71] and kaurenoic acid [72,73,74]. *C. multijuga* [74] and *C. paupera* [75] resins are also good sources of copalic acid.

## 6. Biological Activities of *Copaifera*

*Copaifera* oleoresins have shown remarkable biological activities, many of which have been attributed to diterpenoid acids (see Table 3). Generally, *Copaifera* oleoresins and their diterpenoid constituents have shown antibacterial, anti-inflammatory, antileishmanial, antiproliferative, antitrypanosomal, and wound-healing activities.

### 6.1. Antiparasitic Activity of Copaiba

Several *Copaifera* oleoresin oils have shown in vitro antiparasitic activity against *Leishmania amazonensis* promastigotes, including *C. cearensis*, *C. langsdorffii*, *C. lucens*, *C. martii*, *C. multijuga*, *C. officinalis*, *C. paupera*, and *C. reticulata* [48]. The resin oil of *C. martii* showed in vivo antileishmanial activity in a mouse model [97] and *C. reticulata* resin oil showed activity against *L. amazonensis* axenic amastigotes (IC_50_ = 15.0 μg/mL) and intracellular amastigotes (IC_50_ = 20 μg/mL) [109]. Diterpenoids isolated from *C. officinalis*—agathic acid, alepterolic acid, kaurenoic acid, methyl copalate, pinifolic acid, and *ent*-polyalthic acid—showed antileishmanial activity against *L. amazonensis* promastigotes [104].

*Copaifera* oleoresins and diterpene acids have also shown antitrypanosomal activities. *C. duckei* and *C. reticulata* resins showed in vitro activity against *T. evansi* trypomastigotes [86]. The diterpene acids—agathic acid, copalic acid, alepterolic acid, kaurenoic acid, methyl copalate, pinifolic acid, and *ent*-polyalthic acid—all showed antitrypanosomal activity against *T. cruzi*, including in the epimastigote, trypomastigote, and amastigote forms of the protozoan [111].

A number of parasitic protozoal proteins have been identified as potential targets for antiparasitic chemotherapy [113]. In conjunction with this review, we have examined the potential parasitic targets of *Copaifera* diterpenoids using molecular docking. It is currently not known what biomolecular targets from *Leishmania* or *Trypanosoma* may be responsible for the antiprotozoal activities of copaiba. The *Copaifera* diterpenoids (Figure 1, Figure 2 and Figure 3) were screened, in silico, against *Leishmania* drug targets [114,115,116] and *Trypanosoma cruzi* protein targets [117] using Molegro Virtual Docker v. 6.0.1 as previously described [114,115,116,117]. The docking energies are summarized in Table 4 and Table 5.

The *Leishmania* protein target with the best overall docking properties with *Copaifera* diterpenoids was *L. major* dihydroorotate dehydrogenase (average *E*_dock_ = −109.2 kJ/mol). These docking energies were better than the docking energy for the normal substrate, dihydroorotate (*E*_dock_ = −72.1 kJ/mol) and comparable to the co-crystallized ligand for this protein, nitroorotate (*E*_dock_ = −104.2 kJ/mol). Docking energies for *Copaifera* diterpenoids with TcDHODH (average −92.5 kJ/mol) were also better than the normal substrate (dihydroorotate, *E*_dock_ = −64.2 kJ/mol), but worse than the synthetic TcDHODH inhibitor, 5-[2-(5-carboxynaphthalen-2-yl)ethyl]-2,6-dioxo-1,2,3,6-tetrahydro-pyrimidine-4-carboxylic acid (TT2-2-199, *E*_dock_ = −140.7 kJ/mol). Similarly, *Copaifera* diterpenoids docked with *L. donovani* DHODH (average *E*_dock_ = −89.9 kJ/mol) better than dihydroorotate (*E*_dock_ = −60.9 kJ/mol). Based on these docking energies, protozoal dihydroorotate dehydrogenases are likely targets for *Copaifera* diterpenoids.

*Leishmania major* methionyl-tRNA synthetase was another *Leishmania* protein target with good docking energies. Although the docking energies with this protein were excellent (average *E*_dock_ = −106.9 kJ/mol), they are much poorer than the docking energy of the normal substrate, methionyl adenylate (*E*_dock_ = −168.1 kJ/mol). Similarly, the *T. cruzi* target protein with the best docking was UDP-galactose mutase (average *E*_dock_ = −104.5 kJ/mol), but the normal substrate and co-crystallized ligand, uridine diphosphate (UDP), had a much superior docking energy (*E*_dock_ = −232.8 kJ/mol). Likewise, *L. major* UDP-glucose pyrophosphorylase showed an average docking energy of −99.9 kJ/mol, which was much worse than UDP itself (*E*_dock_ = −145.9 kJ/mol). The diterpenoids showed good docking to *T. cruzi* spermidine synthase, with an average docking energy of −96.8 kJ/mol; however, these are much worse than the docking energy of the co-crystallized ligand, *S*-adenosyl methionine, with a docking energy of −133.0 kJ/mol. Thus, although they exhibited good docking properties, it is unlikely that *Copaifera* diterpenoids can compete with the normal substrate ligands for these proteins.

*Copaifera* diterpenoids showed excellent docking to *L. mexicana* pyruvate kinase (average *E*_dock_ = −103.4 kJ/mol), much better than the normal substrate, phosphoenolpyruvate (*E*_dock_ = −59.8 kJ/mol). Docking energies with *T. cruzi* pyruvate kinase were not as impressive (average −80.3 kJ/mol), but still better than phosphoenolpyruvate (*E*_dock_ = −48.6 kJ/mol) and comparable to the TcPYK inhibitor, ponceau S (*E*_dock_ = −83.6 kJ/mol). Parasite pyruvate kinases can be expected to be target proteins for *Copaifera* diterpenoids.

Protozoal triosephosphate isomerases (LmexTIM and TcTIM) are expected to be targeted by *Copaifera* diterpenoids. The average docking energy with LmexTIM (−90.7 kJ/mol) was much better than either the normal substrate (dihydroxyacetone phosphate, *E*_dock_ = −52.4 kJ/mol) or the co-crystallized ligand, phosphoglycolohydroxamic acid (*E*_dock_ = −61.1 kJ/mol). Likewise, docking energies with TcTIM (average −88.2 kJ/mol) were better than the dihydroxyacetone phosphate (*E*_dock_ = −59.7 kJ/mol) and comparable to the TcTIM inhibitor, 3-(2-benzothiazolylthio)-1-propanesulfonic acid (*E*_dock_ = −85.5 kJ/mol).

Both *L. major* pteridine reductase and *T. cruzi* pteridine reductase had docking properties with *Copaifera* diterpenoids with comparable energies (average *E*_dock_ = −93.8 and −96.8 kJ/mol, respectively) with the normal substrate dihydrobiopterin (*E*_dock_ = −96.9 and −100.1 kJ/mol, respectively). Thus, *Copaifera* diterpenoids may compete with dihydrobiopterin for pteridine reductase.

Sterols are the normal substrates for sterol 14α-demethylase (CYP51), and triterpenoids are expected to also target this protein as inhibitors [118]. Nevertheless, *Copaifera* diterpenoids showed docking energies that may compete with normal sterols for these protein targets. *L. infantum* CYP51 had an average docking energy with the diterpenoids of −90.2 kJ/mol, which was generally not as good as a normal sterol substrate (obtusifoliol, *E*_dock_ = −104.4 kJ/mol), but comparable to the known LinfCYP51 inhibitor fluconazole (*E*_dock_ = −87.5 kJ/mol). Likewise, *T. cruzi* CYP51 had an average diterpenoid docking energy of −89.5 kJ/mol, but substrate (obtusifoliol) docking of −105.6 kJ/mol, and fluconazole docking energy of −90.9 kJ/mol.

*Copaifera* diterpenoids generally showed weak docking energies against the parasite cysteine proteases, *L. donovani* cathepsin B, *L. major* cathepsin B, or cruzain. This docking behavior of diterpenoids with *Leishmania* cathepsin B [114] and cruzain [117] was previously observed. *Leishmania donovani* and *T. cruzi* cyclophilins also showed weak docking energies.

Although *Copaifera* diterpenoids showed only weak docking to parasite glyceraldehyde-3-phosphate dehydrogenases, they may still target these proteins. LmexGAPDH had an average *E*_dock_ of −73.0 kJ/mol and TcGAPDH had an average *E*_dock_ of −70.3 kJ/mol, but these docking energies are better than the docking energies of the normal substrate, glyceraldehyde-3-phosphate (*E*_dock_ = −58.9 and −52.6 kJ/mol, respectively).

Additional *Leishmania* proteins expected to be targeted by *Copaifera* diterpenoids include glycerol-3-phosphate dehydrogenase, which showed excellent docking energies (average −100.4 kJ/mol) to LmexGPDH, better than the normal substrate, glycerol-3-phosphate (*E*_dock_ = −62.5 kJ/mol). Also targeted with a weak docking energy are: glucose-6-phosphate isomerase (Lmex GPI *E*_dock_ average = −73.0 kJ/mol), though better than the docking energy of the normal substrate (glucose-6-phosphate, *E*_dock_ = −62.0 kJ/mol); and phosphomannomutase (LmexPMM *E*_dock_ average = −94.2 kJ/mol), which is better compared to the normal substrate (mannose-6-phosphate, *E*_dock_ = −72.5 kJ/mol).

Additional *T. cruzi* protein targets may be dihydrofolate reductase—thymidylate synthase (TcDHFR–TS), which showed an average docking energy with *Copaifera* diterpenoids of −93.2 kJ/mol, comparable to the docking energy of the normal substrate (dihydrofolate, −99.3 kJ/mol), as well as the TcDHFR–TS inhibitor cycloguanil (*E*_dock_ = −83.1 kJ/mol); farnesyl diphosphate synthase (TcFPPS), with docking energies that averaged −96.2 kJ/mol, which is comparable to the docking energy of the normal substrate, isopentenyl diphosphate (*E*_dock_ = −98.9 kJ/mol); and hypoxanthine phosphoribosyltransferase (TcHPRT), having an average *E*_dock_ = −82.1 kJ/mol, compared to the normal substrate, hypoxanthine, with *E*_dock_ of −65.9 kJ/mol.

### 6.2. Antibacterial Activity of Copaiba

Copaiba oleoresin has shown antibacterial activity against several strains, in particular, Gram-positive *Bacillus subtilis* and *Staphylococcus aureus* with minimum inhibitory concentration (MIC) values of 5 μg/mL for both organisms [110]. Copalic acid, isolated from *C. langsdorffii*, showed excellent antibacterial activity against *Bacillus cereus* (MIC 8.0 μg/mL), *B. subtilis* (MIC 5.0 μg/mL), *Kocuria rhizophila* (MIC 5.0 μg/mL), *Streptococcus pyogenes* (MIC 3.0 μg/mL), *S. pneumoniae* (MIC 3.0 μg/mL), *S. agalactiae* (MIC 2.0 μg/mL), *S. dysgalactiae* (MIC 1.0 μg/mL), *S. epidermidis* (MIC 0.5 μg/mL) [71], *S. salivarius* (MIC 2.0 μg/mL), *S. mutans* (MIC 3.0 μg/mL), *S. mitis* (MIC 5.0 μg/mL), *S. sobrinus* (MIC 3.0 μg/mL), and *S. sanguinis* (MIC 6.0 μg/mL) [70]. Likewise, kaurenoic acid showed remarkable activity against *S. pyogenes* (MIC 5.0 μg/mL), *S. pneumoniae* (MIC 5.0 μg/mL), *S. dysgalactiae* (MIC 8.0 μg/mL) [71], *S. epidermidis* (MIC 4–5 μg/mL), *B. subtilis* (MIC 3.1–6.3 μg/mL), and *S. aureus* (MIC 8–10 μg/mL) [75]. 3α-Alepterolic acid acetate (acetoxycopalic acid) showed moderate antibacterial activity against cariogenic *Streptococcus* bacteria, with MIC values ranging from 12.0 to 60.0 μg/mL [70]. *ent*-Polyalthic acid also showed moderate antibacterial activity against *B. subtilis* (MIC 20–30 μg/mL), *S. aureus* (MIC 40–50 μg/mL), and *S. epidermidis* (MIC 40 μg/mL) [75].

In order to provide some insight into the mechanisms of activity, a virtual screening of copaiba diterpenoids has been carried out against several bacterial protein targets, including peptide deformylase, DNA gyrase, topoisomerase IV, UDP-galactopyranose mutase, protein tyrosine phosphatase, cytochrome P450 CYP 121, and nicotinamide adenine dinucleotide (NAD^+^)-dependent DNA ligase [119] (see Table 6). The best bacterial target for copalic acid was *Mycobacterium tuberculosis* DNA gyrase B (PDB 3ZKD) with a docking energy (*E*_dock_) of −105.7 kJ/mol. The protein with the best docking energy with kaurenoic acid was *S. pneumoniae* peptide deformylase (PDB 2AIE, *E*_dock_ = −89.7 kJ/mol). 3α-Alepterolic acid acetate was the best docking ligand to *Escherichia coli* topoisomerase IV (PDB 1S16) and *M. tuberculosis* DNAGyrB (PDB 3ZKD) with docking energies of −118.8 and −118.3 kJ/mol, respectively. 3β-Alepterolic acid acetate also showed excellent docking to these two proteins with docking energies of −117.1 and −117.3 kJ/mol, respectively. The best bacterial target for *ent*-polyalthic acid was *M. tuberculosis* protein tyrosine phosphatase (PDB 2OZ5, *E*_dock_ = −107.2 kJ/mol). The copaiba diterpenoid ligand with the best docking properties was 7α-acetoxyhardwickiic acid with *S. aureus* peptide deformylase (PDB 3U7M, *E*_dock_ = −120.6 kJ/mol).

### 6.3. Antiproliferative Activity of Copaiba

Copaiba oleoresins have exhibited both in vitro and in vivo antiproliferative activities (Table 3). *Copaifera reticulata* oleoresin, for example, has shown in vitro cytotoxic activity against GM07492-A human lung fibroblast cells with an IC_50_ of 51.85 μg/mL [68]. The oleoresin of *C. multijuga* has shown in vitro cytotoxic activity against B16F10 murine melanoma cells with an IC_50_ of 457 μg/mL [57]. Furthermore, in a mouse model of lung metastasis and tumor growth, oral administration of *C. multijuga* oleoresin reduced tumor growth, tumor mass, and number of lung nodules after inoculation of B16F10 tumor cells [57]. Likewise, *C. multijuga* oleoresin, in doses varying between 100 and 200 mg/kg, showed antineoplastic properties against Ehrlich ascetic tumors and solid tumors in an in vivo mouse model [98]. On the other hand, *C. officinalis* oleoresin actually stimulated growth of Walker 256 carcinoma by 70% in an in vivo rat model [101].

Diterpenoids isolated from *Copaifera* species have shown cytotoxic activities (Table 3). Copalic acid, isolated from *C. langsdorffii*, showed in vitro cytotoxicity on MO59J human glioblastoma cells and HeLa human cervical adenocarcinoma cells with IC_50_ of 68.3 and 44.0 μg/mL, respectively [71]. Kaurenoic acid has demonstrated cytotoxicity against several human tumor cell lines, including CEM leukemia, MCF-7 breast tumor, HCT-8 colon tumor [73], AGP01 gastric tumor, and SF-295 glioblastoma [74]. Growth inhibition of AGP01 and SF-295 cells was also demonstrated by 3β-alepterolic acid and 3β-alepterolic acid acetate [73]. Methyl copalate showed remarkable cytotoxic activity on P-388 murine lymphoma (IC_50_ = 2.5 μg/mL), A-549 human lung carcinoma (IC_50_ = 5 μg/mL), HT-29 human colon carcinoma (IC_50_ = 5 μg/mL), and MEL-28 human melanoma (IC_50_ = 10 μg/mL) cells [75].

Molecular docking (Molegro Virtual Docker, Aarhus, Denmark) has been carried out with *Copaifera* diterpenoids on cancer molecular targets, including androgen receptor, aromatase, caseine kinase II, cyclin-dependent kinases 2, 4, and 6, cyclooxygenase 2, DNA (cytosine-5)-methyltransferase-1 and -3A, epidermal growth factor receptor, estrogen receptor α, estrogen receptor β, heat shock protein 90, insulin-like growth factor 1 receptor, 5-lipoxygenase, mitogen-activated protein kinase 1, NF-κB, p90 ribosomal protein S6 kinase, P-glycoprotein, phosphatidylinositol-4,5-bisphosphate 3-kinase, topoisomerase I, topoisomerase IIα, topoisomerase IIβ, tubulin, and vascular endothelial growth factor receptor (Table 7). The best overall cancer targets for the copaiba diterpenoids were human DNA (cytosine-5)-methyltransferase-1 (HsDNMT1), human estrogen receptor β (HsERβ), and human mitogen-activated protein kinase 1 (HsMEK1), with average MolDock docking energies of −102.7, −99.2, and −101.5 kJ/mol, respectively. DNA (cytosine-5)-methyltransferase-1 (DNMT1) is the enzyme responsible for DNA methylation of carbon-5 of cytosine within CpG dinucleotides [120]. The enzyme is required for embryonic development [121], but is overexpressed in lung, liver, colorectal, gastric, breast, and lung tumors [122]. Thus, DNMT1 has emerged as an attractive target for cancer chemotherapy [123,124]. The mitogen-activated protein kinase (MAPK) signaling cascade is one of the most important pathways involved in cellular proliferation and differentiation [125] and, therefore, inhibition of components of this pathway, such as MEK1, can potentially target tumors that depend on MAPK signaling [126]. Agonism of estrogen receptor α (ERα) stimulates proliferation of breast, uterus, and prostate tissues, whereas ERβ agonism inhibits proliferation of these tissues [127]. Thus, compounds that can selectively bind and activate ERβ, but not ERα, could represent effective antitumor agents for treatment of prostate and breast cancer [128]. Copalic acid and methyl copalate both targeted HsMEK1, with docking energies of −108.2 and −111.0 kJ/mol, respectively, while 3β-alepterolic acid and 3β-alepterolic acid acetate showed excellent docking with HsDNMT1 (*E*_dock_ = −107.2 and −121.7 kJ/mol, respectively). Kaurenoic acid was a relatively weakly docking ligand but did show selective docking to aromatase (*E*_dock_ = −93.7 kJ/mol). The best-docking ligand was patagonic acid, which had a docking energy of −121.8 kJ/mol with HsDNMT1.

### 6.4. Anti-Inflammatory Activity of Copaiba

Inflammation is the biological response of body tissues to detrimental stimuli, such as pathogenic microorganisms, chemical or physical irritants, or injury. Inflammation is manifested by redness, swelling, heat, and sometimes pain. While acute inflammation is a normal part of the healing process, chronic inflammation often plays a role in chronic diseases such as osteoarthritis, lupus, and inflammatory bowel disease, and can be problematic. Several copaiba oleoresins have shown anti-inflammatory activity, including *C. cearensis* [13], *C. duckei* [84], *C. langsdorffii* [50,91], *C. multijuga* [13,58,61,100], *C. officinalis* [62], and *C. reticulata* [13] (Table 3).

The immune response is a complex cascade of interacting cytokines and reactions, and there are several biomolecular targets important in treating chronic inflammation. We have carried out virtual screening of copaiba diterpenoids against soluble epoxide hydrolase (EPHX2), fibroblast collagenase, phospholipase A2 (PLA2), 5-lipoxygenase, inducible nitric oxide synthase, phosphoinositide 3-kinase, interleukin-1 receptor-associated kinase 4, glutathione *S*-transferase ω-1, cyclooxygenase-1, cyclooxygenase-2, c-Jun *N*-terminal kinase, nuclear factor κ-light-chain-enhancer of activated B cells (NF-κB), inhibitor of κB kinase β, NF-κB essential modulator, lipid binding protein MD-2, myeloperoxidase, p38 mitogen-activated protein kinase, peroxisome proliferator-activated receptor γ, and cAMP-specific 3′,5′-cyclic phosphodiesterase 4D (Table 8). The overall best target proteins were murine soluble epoxide hydrolase and murine phospholipase A2, with average docking energies of −108.3 and −100.0 kJ/mol. Secretory phospholipase A2 and cytosolic phospholipase A2 are both targets for anti-inflammatory drug development [129]. Soluble epoxide hydrolase has been identified as a molecular target not only for inflammatory diseases, but also as a target for neurodegenerative diseases and for treatment of pain [130]. Thus, targeting EPHX2 and/or PLA2 by copaiba diterpenoids may explain the anti-inflammatory activities of copaiba oleoresins.

## 7. Computational Methods—Molecular Docking

Molecular docking analyses were carried out using Molegro Virtual Docker (v. 6.0.1, Molegro ApS, Aarhus, Denmark) against known bacterial [119], *Leishmania* [114,115,116], *Trypanosoma cruzi* [117], and cancer-relevant and inflammation-relevant protein targets [131], as previously described [114,115,116,117,119,132].

## 8. Conclusions

The oleoresins from *Copaifera* species (copaiba) have been used by native peoples of the Amazon region for thousands of years. These materials have shown remarkable biological activities, including antibacterial, antiparasitic, antineoplastic, and anti-inflammatory activities. Copaiba resins have been distilled to give essential oils that are largely composed of sesquiterpenoids, particularly β-caryophyllene. The resins are also composed of diterpene acids, which are responsible for many of the observed biological activities. Molecular docking of copaiba diterpene acids with documented protein targets has revealed potential mechanisms of activity for these bioactive constituents. Future research to validate the molecular mechanisms of copaiba diterpenoids is encouraged.

## Figures and Tables

**Table 1:** Major components of the essential oils of *Copaifera* species.

*Copaifera* spp.	EO Source	Major Components (>5%)	Ref.
*C. cearensis* Huber ex Ducke	oleoresin	α-copaene (8.2%), β-caryophyllene (19.7%), δ-cadinene (7.2%), β-bisabolol (8.2%), hardwickiic acid (6.2%), clorechinic acid (11.3%)	[13]
*C. cearensis* Huber ex Ducke	oleoresin	α-copaene (8.2%), β-caryophyllene (19.7%), hardwickiic acid (6.2%)	[48]
*C. duckei* Dwyer	oleoresin	β-caryophyllene (0.7–6.2%), *trans*-α-bergamotene (3.4–7.9%), β-selinene (5.5–7.3%), β-bisabolene (8.9–12.1%), kaur-16-en-19-oic acid (19.8–24.5%), polyalthic acid (17.1–27.7%), hardwickiic acid (0–24.3%)	[8]
*C. duckei* Dwyer	oleoresin	β-elemene (8.3–9.4%), β-caryophyllene (13.0–15.5%), *trans*-α-bergamotene (8.3–10.6%), β-selinene (13.8–15.4%), α-selinene (8.8–9.9%), β-bisabolene (15.7–17.6%)	[49]
*C. duckei* Dwyer	oleoresin	β-caryophyllene (25.1–50.2%), *trans*-α-bergamotene (6.4–12.0%), (*E*)-β-farnesene (2.9–5.8%), β-selinene (1.8–6.7%), β-bisabolene (5.2–33.6%)	[49]
*C. guianensis* Desf.	oleoresin	*trans*-α-bergamotene (7.2%), caryophyllene oxide (19.1%), kaur-16-en-19-oic acid (17.5%), polyalthic acid (10.6%), hardwickiic acid (11.0%)	[8]
*C. langsdorffii* Desf.	oleoresin	β-caryophyllene (32.8%), kaurenoic acid (44.3%), copalic acid (5.6%), hardwickiic acid (8.2%)	[48]
*C. langsdorffii* Desf.	oleoresin	cyclosativene (5.0%), β-elemene (5.1%), β-caryophyllene (5.5%), *trans*-α-bergamotene (48.4%), β-selinene (5.0%), α-himachalene (11.2%)	[50]
*C. langsdorffii* Desf.	oleoresin	α-copaene (5.8%), γ-muurolene (22.7%), eremophilone (6.8%), kaurene (6.8%), methyl oleate (26.5%)	[51]
*C. langsdorffii* Desf.	oleoresin	β-elemene (8.0%), β-caryophyllene (31.4%), *trans*-α-bergamotene (10.2%), γ-muurolene (16.1%)	[52]
*C. langsdorffii* Desf.	oleoresin	β-caryophyllene (1.1–9.0%), germacrene D (4.0–18.0%), bicyclogermacrene (1.5–5.7%), spathulenol (12.6–35.7%), caryophyllene oxide (7.4–16.6%), α-cadinol (3.2–7.9%)	[53]
*C. langsdorffii* Desf.	pericarp	α-copaene (3.2–14.4%), β-elemene (0–11.1%), β-caryophyllene (2.7–10.5%), germacrene D (1.9–10.7%), bicyclogermacrene (0–6.3%), spathulenol (2.2–16.2%), caryophyllene oxide (4.0–5.1%), *iso*-spathulenol (5.6–21.6%)	[54]
*C. langsdorffii* Desf.	leaf	α-copaene (1.8–6.9%), β-elemene (0–8.4%), β-caryophyllene (5.7–17.5%), germacrene D (0–17.3%), bicyclogermacrene (0–11.5%), δ-cadinene (1.6–6.1%), spathulenol (3.8–12.4%), caryophyllene oxide (0–15.6%), α-muurolol (4.8–6.2%), α-cadinol (4.9–6.8%)	[54]
*C. langsdorffii* Desf.	leaf	β-caryophyllene (10.1–16.8%), germacrene D (9.1–45.2%), bicyclogermacrene (4.8–21.1%), spathulenol (4.9–29.4%), caryophyllene oxide (3.8–18.8%)	[55]
*C. langsdorffii* Desf.	seed	coumarin (0–12.6%), spathulenol (19.4–38.9%), caryophyllene oxide (0–21.8%), humulene epoxide II (0–5.1%), *iso*-spathulenol (6.9–25.8%), τ-muurolol (1.3–5.0%), α-cadinol (2.0–10.4%)	[54]
*C. langsdorffii* Desf.	stem	β-caryophyllene (2.4–13.9%), germacrene D (0–19.1%), bicyclogermacrene (0–8.0%), δ-cadinene (0–5.7%), spathulenol (3.6–13.7%), caryophyllene oxide (4.9–13.3%), *iso*-spathulenol (0–7.9%), τ-muurolol (3.4–7.9%), α-cadinol (4.9–11.5%)	[54]
*C. lucens* Dwyer	oleoresin	polyalthic acid (69.8%), copalic acid (11.1%)	[48]
*C. martii* Hayne	oleoresin	α-copaene (36.4–51.2%), β-elemene (4.1–6.2%), *allo*-aromadendrene (4.2–5.0%), δ-cadinene (13.7–17.2%)	[56]
*C. martii* Hayne	oleoresin	β-bisabolene (10.7%), zingiberene (7.2%), kaurenoic acid (7.9%), kovalenic acid (29.0%)	[48]
*C. multijuga* Hayne	oleoresin	α-copaene (2.1–5.2%), β-caryophyllene (42.9–60.3%), *trans*-β-bergamotene (2.0–7.0%), caryophyllene oxide (tr–8.8%), copalic acid (1.9–11.0%), 3-acetoxycopalic acid (0.8–6.2%)	[8]
*C. multijuga* Hayne	oleoresin	β-caryophyllene (57.5%), α-humulene (8.3%), copalic acid (6.2%)	[57]
*C. multijuga* Hayne	oleoresin	β-caryophyllene (57.5%), α-humulene (8.3%), copalic acid (6.2%)	[13]
*C. multijuga* Hayne	oleoresin	β-caryophyllene (60.2%), *trans*-α-bergamotene (6.4%), α-humulene (8.6%), copalic acid (9.5%)	[44]
*C. multijuga* Hayne	oleoresin	β-caryophyllene (57.5%), copalic acid (6.2%)	[48]
*C. multijuga* Hayne	oleoresin	α-copaene (18.8%), β-caryophyllene (36.0%), *trans*-α-bergamotene (7.0%), β-bisabolene (8.5%), δ-cadinene (6.1%)	[58]
*C. multijuga* Hayne	oleoresin	α-copaene (2.0–15.0%), β-caryophyllene (5.1–64.0%), α-humulene (0–8.9%), germacrene D (0–16.7%), δ-cadinene (0–5.4%), caryophyllene oxide (0.2–31.5%), copalic acid (1.7–7.1%)	[17]
*C. multijuga* Hayne	oleoresin	β-caryophyllene (57.1%), α-humulene (10.2%), β-sesquiphellandrene (9.9%)	[59]
*C. multijuga* Hayne	oleoresin	α-copaene (2.5–14.9%), β-caryophyllene (10.6–62.7%), α-humulene (2.4–8.7%), germacrene D (0–18.9%), caryophyllene oxide (0.2–32.5%), copalic acid (1.1–5.2%)	[60]
*C. multijuga* Hayne	oleoresin	α-copaene (5.0%), β-gurjunene (5.3%), β-caryophyllene (29.6%), α-humulene (5.7%), caryophyllene alcohol (5.8%), caryophyllene oxide (13.0%)	[61]
*C. multijuga* Hayne	oleoresin	β-caryophyllene (58.4%), α-humulene (8.4%), copalic acid (6.3%)	[61]
*C. officinalis* (Jacq.) L.	oleoresin	β-caryophyllene (8.5%), copalic acid (13.9%), hardwickiic acid (30.7%)	[48]
*C. officinalis* (Jacq.) L.	oleoresin	β-caryophyllene (24.9%), *allo*-aromadendrene (7.5%), germacrene B (5.1%), β-bisabolene (6.3%), δ-cadinene (15.3%), α-cadinene (5.6%)	[62]
*C. officinalis* (Jacq.) L.	oleoresin	β-caryophyllene (62.7%), *trans*-α-bergamotene (7.6%), α-humulene (8.1%)	Setzer ^a^
*C. officinalis* (Jacq.) L.	oleoresin	β-caryophyllene (87.3%)	Setzer ^b^
*C. paupera* (Herzog) Dwyer	oleoresin	β-bisabolene (20.2%), zingiberene (19.4%), kaurenoic acid (13.3%), copalic acid (6.1%)	[48]
*C. paupera* (Herzog) Dwyer	oleoresin	α-cubebene (5.5%), α-copaene (42.5%), β-caryophyllene (14.1%), δ-cadinene (10.4%)	[63]
*C. piresii* Ducke	oleoresin	α-copaene (45.5%), β-caryophyllene (10.3%), δ-cadinene (13.7%)	[63]
*C. publflora* Benth.	oleoresin	β-caryophyllene (65.9%), α-humulene (7.3%), β-selinene (10.2%), α-selinene (5.5%)	[63]
*C. reticulata* Ducke	oleoresin	β-caryophyllene (40.9%), α-humulene (6.0%), germacrene D (5.0%)	[13]
*C. reticulata* Ducke	oleoresin (Pará)	β-caryophyllene (40.9%)	[48]
*C. reticulata* Ducke	oleoresin (Acre)	α-copaene (25.1%), β-caryophyllene (13.1%), copalic acid (7.7%), kaurenoic acid (7.5%), hardwickiic acid (6.9%)	[48]
*C. reticulata* Ducke	oleoresin	β-elemene (0.5–5.6%), β-caryophyllene (1.4–68.0%), *trans*-α-bergamotene (2.4–29.6%), α-humulene (1.1–9.7%), β-selinene (0–20.6%), α-selinene (0–13.2%), β-bisabolene (3.7–42.4%), caryophyllene oxide (0.1–15.2%)	[64]
*C. reticulata* Ducke	oleoresin	β-elemene (0–6.0%), β-caryophyllene (0–43.4%), *trans*-α-bergamotene (12.0–32.8%), α-guaiene (0–9.5%), α-humulene (0–7.0%), β-selinene (0–17.1%), α-selinene (0–10.4%), *trans*-β-guaiene (0–5.8%), β-bisabolene (24.2–50.3%)	[65]
*C. reticulata* Ducke	oleoresin	β-caryophyllene (25.1–50.2%), *trans*-α-bergamotene (6.4–12.0%), α-humulene (4.1–5.8%), β-selinene (1.8–6.7%), β-bisabolene (5.2–17.4%)	[66]
*C. reticulata* Ducke	oleoresin	β-caryophyllene (37.3%), *trans*-α-bergamotene (9.0%), α-humulene (5.4%), β-bisabolene (14.5%)	[67]
*C. reticulata* Ducke	oleoresin	β-caryophyllene (7.7%), *trans*-α-bergamotene (22.0%), β-selinene (12.2%), α-selinene (11.4%), β-bisabolene (24.9%)	[68]
*C. trapezifolia* Hayne	leaf	β-caryophyllene (33.5%), α-humulene (6.2%), germacrene D (11.0%), spathulenol (7.6%)	[69]

^a^ Unpublished analysis of a commercial essential oil from New Directions Aromatics (Sydney, Australia). ^b^ Unpublished analysis of a commercial essential oil from Améo Essential Oils/Zija International (Lehi, Utah).

**Table 2:** Nonvolatile components isolated and/or identified from *Copaifera* species.

*Copaifera* spp.	Plant Part	Compounds Isolated and/or Identified	Ref.
*C. cearensis* Huber ex Ducke	oleoresin	epuric acid, cativic acid, copalic acid, kolavenic acid, crolechinic acid, hardwickiic acid, haplociliatic acid, labdanolic acid, patagonic acid.	[76]
*C. guianensis* Desf.	oleoresin	methyl copalate	[74]
*C. langsdorffii* Desf.	oleoresin	copalic acid, kaurenoic acid, acetoxycopalic acid (=3α-alepterolic acid acetate), *ent*-agathic acid, hydroxycopalic acid (=3α-alepterolic acid)	[71]
*C. langsdorffii* Desf.	oleoresin	copalic acid, acetoxycopalic acid (=3α-alepterolic acid acetate), 3-hydroxy-14,15-dinorlabd-8(17)-en-13-one, *ent*-agathic acid, hydroxycopalic acid (=3α-alepterolic acid)	[70]
*C. langsdorffii* Desf.	oleoresin	kaurenoic acid	[72]
*C. langsdorffii* Desf.	oleoresin	kaurenoic acid	[73]
*C. langsdorffii* Desf.	oleoresin	kaurenoic acid	[74]
*C. langsdorffii* Desf.	oleoresin	sclareol, manool, copalic acid, acetoxycopalic acid (=3α-alepterolic acid acetate), hydroxycopalic acid (=3α-alepterolic acid), *ent*-agathic acid	[77]
*C. langsdorffii* Desf.	leaves	kaurenoic acid, quercitrin, afzelin	[78]
*C. langsdorffii* Desf.	leaves	rutin, quercetin-3-*O*-α-l-rhamnopyranoside, kaempferol-3-*O*-α-l-rhamnopyranoside, quercetin, kaempferol	[79]
*C. langsdorffii* Desf.	fruit	gallic acid, epicatechin gallate, catechin, epicatechin, isoquercitrin	[80]
*C. langsdorffii* Desf.	leaves	kaurenoic acid, quercitrin, afzelin, eupatorin, galloyl quinic acid, gallic acid 4-*O*-glucoside	[81]
*C. multijuga* Hayne	oleoresin	copalic acid, 3-hydroxycopalic acid (=3α-alepterolic acid), 3-acetoxycopalic acid (=3α-alepterolic acid acetate)	[74]
*C. paupera* (Herzog) Dwyer	oleoresin	copalic acid, methyl copalate, agathic acid 15-methyl ester, agathic acid 15,19-dimethyl ester, *ent*-polyalthic acid, methyl *ent*-polyalthate, *ent*-pinifolic acid, methyl 3β-hydroxy-labda-8(17),13-dien-15-ate, methyl 18-hydroxy-copaiferolate, 14,15-bisnorlabd-8(17)-en-13-one, *ent*-kaurenic acid, 16β-kauran-19-oic acid, 3-methyl-5-(2,2,6-trimethyl-6-hydroxycyclohexyl)- pentanoic acid, pauperol	[75]
*C. reticulata* Ducke	oleoresin	3β-alepterolic acid, 3β-alepterolic acid acetate, 3β-hydroxylabdan-8(17)-en-15-oic acid, *ent*-agathic acid	[82]

**Table 3:** Biological activities of *Copaifera* oleoresins, essential oils, and isolated components.

*Copaifera* spp.	Material	Biological Activity	Ref.
*C. cearensis* Huber ex Ducke	oleoresin	Anti-inflammatory: At a concentration of 50 μg/mL, in vitro NO production in mouse peritoneal macrophages was significantly reduced by *C. cearensis* oil.	[13]
*C. cearensis* Huber ex Ducke	oleoresin	Antileishmanial: *L. amazonensis* promastigotes (IC_50_ = 18.0 μg/mL).	[48]
*C. cearensis* Huber ex Ducke	oleoresin	Antibacterial: Methicillin-resistant *Staphylococcus aureus* (MRSA, MIC = 125 μg/mL), *B. subtilis* (MIC = 62.5 μg/mL), *Enterococcus faecalis* (MIC = 62.5 μg/mL)	[83]
*C. duckei* Dwyer	oleoresin	Anti-inflammatory: Carrageenin-induced paw edema test (rats, 18% edema inhibition with dose of 1802 mg/kg; granuloma test, 42% inhibition with dose of 1802 mg/kg); croton oil-induced dermatitis test (mice, IC_50_ = 663 mg/kg)	[84]
*C. duckei* Dwyer	oleoresin	Antinociceptive: Mouse model (acetic acid-induced writhing test, IC_50_ = 704 mg/kg)	[84]
*C. duckei* Dwyer	oleoresin	Antiproliferative: In vivo hepatocellular proliferation, partial hepatectomy, rats. Hepatocellular proliferation and liver mitochondrial respiration were significantly lower in *C. duckei* treated rats compared to control (saline solution).	[85]
*C. duckei* Dwyer	oleoresin	Antitrypanosomal: *T. evansi*, in vivo mouse model, doses of 0.63 mL/kg/day over 5 days showed no curative effects. *T. evansi*, in vitro trypomastigotes, 0.5% solution of *C. duckei* oil showed 100% killing after 6 h.	[86]
*C. langsdorffii* Desf.	oleoresin	Anti-inflammatory: Preincubation of LPS-stimulated human THP-1 monocytes with diterpenoid-rich oleoresin reduced the release of proinflammatory cytokines (IL-1β, IL-6, TNFα).	[50]
*C. langsdorffii* Desf.	oleoresin	Antifungal: *Tricophyton mentagrophytes* (MIC = 170 μg/mL). Scanning electron microscopic (SEM) analysis revealed physical damage and morphological alterations of the fungi upon exposure to copaiba oleoresin.	[52]
*C. langsdorffii* Desf.	oleoresin	Antipsoriatic: Human clinical trial, topical application of copaiba resin exhibited a significant improvement of the typical signs of psoriasis.	[50]
*C. langsdorffii* Desf.	oleoresin	Gastroprotective: Ethanol or indomethacin-induced ulcer in rats, oral administration of copaiba resin at doses of 400 mg/kg provided dose-dependent significant protection against gastric damage caused by ethanol or indomethacin.	[87]
*C. langsdorffii* Desf.	oleoresin	Gastroprotective: Mesenteric ischemia/reperfusion (I/R) in rats: Copaiba resin treatment caused significant attenuations in I/R-associated increases of myeloperoxidase, malondialdehyde, and catalase, and effectively prevented the I/R-associated depletion of glutathione.	[88]
*C. langsdorffii* Desf.	oleoresin	Wound-healing: Rat incision wounds, 4% oleoresin topically applied showed significant wound contraction and tensile strength compared to controls.	[89]
*C. langsdorffii* Desf.	oleoresin	Wound healing: Rat dorsal skin flaps, oral administration of copaiba oleoresin (400 mg/kg), copaiba-treatment presented discrete anti-lipoperoxidation action, intense antioxidant action, and anti-inflammatory activity during the ischemia and reperfusion of randomized cutaneous flaps.	[90]
*C. langsdorffii* Desf.	oleoresin	Anti-inflammatory: Rat model of experimental endometriosis. Copaiba oil caused a marked reduction in endometrial growth.	[91]
*C. langsdorffii* Desf.	oleoresin	Antileishmanial: *L. amazonensis* promastigotes (IC_50_ = 20.0 μg/mL).	[48]
*C. langsdorffii* Desf.	oleoresin	Antibacterial: *B. subtilis* (MIC = 62.5 μg/mL)	[83]
*C. langsdorffii* Desf.	10% copaiba oil ointment	Wound-healing: Rat dorsal skin flaps, topical copaiba oil ointment favors angiogenesis and accelerates the viability of random skin flaps in rats.	[51]
*C. langsdorffii* Desf.	10% oleoresin cream	Antibacterial: Open wounds on rats inoculated with *Streptococcus pyogenes* or *Staphylococcus aureus*. Treatment with 10% copaiba cream reduced bacterial populations to 0.02% (*S. pyogenes*) and 0.3% (*S. aureus*) after 14 days.	[92]
*C. langsdorffii* Desf.	10% oleoresin cream	Wound-healing: Rabbit ears, 10% oleoresin cream-treated wounds presented better clinical outcomes, confirmed by histology with evidence of fibroblastic activity by day 7 and organized collagen fibers observed from day 14.	[93]
*C. langsdorffii* Desf.	10% oleoresin cream	Wound-healing: Rat skin biopsy punch on dorsal surface, 10% oleoresin cream-treated wounds showed a faster wound-healing rate compared to saline or cream only controls, by regulating matrix metalloproteinase, (MMP)-2 and MMP-9 activities, stimulating collagen synthesis, and promoting tissue remodeling and re-epithelialization.	[94]
*C. langsdorffii* Desf.	3α-alepterolic acid acetate	Antibacterial: Cariogenic *Streptococcus* spp.; MIC range 12.0–60.0 μg/mL	[70]
*C. langsdorffii* Desf.	aqueous leaf extract	Insecticidal: 5% Extract inhibited *Bemisia tabaci* infestation of tomato plants in the field.	[95]
*C. langsdorffii* Desf.	copalic acid	Antibacterial: Gram-positive bacteria (MIC range 0.5 μg/mL to 15.0 μg/mL)	[71]
*C. langsdorffii* Desf.	copalic acid	Antibacterial: Cariogenic *Streptococcus* spp.; MIC range 2.0–6.0 μg/mL	[70]
*C. langsdorffii* Desf.	copalic acid	Antibacterial: Periodontal anaerobic bacteria: *Actinomyces naeslundii* (MIC 6.2 μg/mL), *Bacteroides fragilis* (MIC 25.0 μg/mL), *Peptostreptococcus anaerobius* (MIC 3.1 μg/mL), *Porphyromonas gingivalis* (MIC 3.1 μg/mL).	[77]
*C. langsdorffii* Desf.	copalic acid	Antiproliferative: In vitro cytotoxicity on MO59J (human glioblastoma cells, IC_50_ = 68.3 μg/mL) and HeLa (human cervical adenocarcinoma cells, IC_50_ = 44.0 μg/mL).	[71]
*C. langsdorffii* Desf.	EtOH/H_2_O leaf extract	Gastroprotective: Ethanol/HCl-induced ulcer in mice, the extract (500 mg/kg) showed a significant decrease in the total gastric juice acidity and an increase in mucus production; isolated compounds (30 mg/kg) α-humulene, β-caryophyllene and caryophyllene oxide showed greater gastroprotective activity in the ethanol/HCl induced ulcer model.	[78]
*C. langsdorffii* Desf.	kaurenoic acid	Anti-inflammatory: Rat model of acetic acid-induced colitis. A marked reduction in gross damage score (52% and 42%) and wet weight of damaged colon tissue (39% and 32%) were observed in rats that received 100 mg/kg kaurenoic acid, respectively, by rectal and oral routes.	[72]
*C. langsdorffii* Desf.	kaurenoic acid	Antibacterial: Gram-positive bacteria (MIC range 5.0 μg/mL to 100.0 μg/mL)	[71]
*C. langsdorffii* Desf.	kaurenoic acid	Antiproliferative: In vitro cytotoxicity, 78 μM concentration, on CEM (human leukemia, 95% growth inhibition), MCF-7 (human breast tumor, 45% growth inhibition), and HCT-8 (human colon tumor, 45% growth inhibition) cells.	[73]
*C. langsdorffii* Desf.	kaurenoic acid	Antiproliferative: In vitro cytotoxicity, 20 μM concentration, on AGP01 (human gastric cancer, 28% growth inhibition) and SF-295 (human glioblastoma, 28% growth inhibition) cells.	[74]
*C. langsdorffii* Desf.	kaurenoic acid	Smooth muscle relaxant: Rat uterine muscle ex vivo: kaurenoic acid, exerts a uterine relaxant effect acting principally through calcium blockade and in part, by the opening of ATP-sensitive potassium channels.	[96]
*C. lucens* Dwyer	oleoresin	Antileishmanial: *L. amazonensis* promastigotes (IC_50_ = 20.0 μg/mL).	[48]
*C. lucens* Dwyer	oleoresin	Antibacterial: *S. aureus* (MIC = 125 μg/mL), *B. subtilis* (MIC = 125 μg/mL)	[83]
*C. martii* Hayne	oleoresin	Antileishmanial: *L. amazonensis* promastigotes (IC_50_ = 14.0 μg/mL).	[48]
*C. martii* Hayne	oleoresin	Antileishmanial: In vivo mouse model, copaiba oil oral treatment (100 mg/kg/day) caused a significant reduction in the average lesion size (1.1 ± 0.4 mm) against *Leishmania amazonensis* lesions compared with untreated mice (4.4 ± 1.3 mm).	[97]
*C. martii* Hayne	oleoresin	Antibacterial: *S. aureus* (MIC = 62.5 μg/mL), MRSA (MIC = 62.5 μg/mL), *B. subtilis* (MIC = 15.6 μg/mL), *S. epidermidis* (MIC = 62.5 μg/mL), *Enterococcus faecalis* (MIC = 62.5 μg/mL)	[83]
*C. multijuga* Hayne	oleoresin	Antiproliferative: In vitro cytotoxicity, B16F10 (murine melanoma cells, IC_50_ = 457 μg/mL).	[57]
*C. multijuga* Hayne	oleoresin	Antiproliferative: In vivo lung metastasis and tumor growth, mouse model: Oral administration of *C. multijuga* oleoresin (at 2 g/Kg in the days 3, 5, 7, 10, 12, and 14 after inoculation of tumoral cells) reduced tumor growth by 58% and tumor weight by 76% and reduced the number of lung nodules by 47.1%.	[57]
*C. multijuga* Hayne	oleoresin	Antiproliferative: In vivo Ehrlich ascitic and solid tumor, mouse model: *C. multijuga* oleoresin (doses varying between 100 and 200 mg/kg) showed antineoplastic properties against Ehrlich ascitic tumor (EAT) and solid tumor during 10 consecutive days of treatment.	[98]
*C. multijuga* Hayne	oleoresin	Insecticidal: Mosquito larvicidal activity (*Anopheles darlingi*, LC_50_ = 31 μg/mL; *Aedes aegypti*, LC_50_ = 93 μg/mL)	[59]
*C. multijuga* Hayne	oleoresin	Anti-inflammatory: The β-caryophyllene-rich oleoresin oil of *C. multijuga*, at a dose of 100 mg/kg, inhibited zymosan-induced pleurisy in a mouse model, reducing total leukocytes by 45% and neutrophil accumulation by 73%. *C. multijuga* oil also showed in vitro reduction of NO production in mouse peritoneal macrophages at a concentration of 5 μg/mL.	[13]
*C. multijuga* Hayne	oleoresin	Anti-inflammatory: Rat pleurisy model, doses of 100 mg/kg and 200 mg/kg presented in vivo anti-inflammatory effects.	[58]
*C. multijuga* Hayne	oleoresin	Antileishmanial: *L. amazonensis* promastigotes (IC_50_ = 10.0 μg/mL).	[48]
*C. multijuga* Hayne	oleoresin	Antibacterial: MRSA (MIC = 125 μg/mL), *B. subtilis* (MIC = 125 μg/mL)	[83]
*C. multijuga* Hayne	oleoresin	Antinociceptive: Mouse model (acetic acid-induced writhing, tail flick, hot plate), oral administration of *C. multijuga* oil with doses of 30–150 mg/kg significantly showed antinociception, which was reversed with naloxone.	[99]
*C. multijuga* Hayne	oleoresin	Insecticidal: Mosquito larvicidal activity (*Anopheles darlingi*, LC_50_ = 128 μg/mL; *Aedes aegypti*, LC_50_ = 18 μg/mL)	[59]
*C. multijuga* Hayne	oleoresin extracts	Anti-inflammatory: The CH_2_Cl_2_ and MeOH fractions obtained from *C. multijuga* oleoresin, given by the intraperitoneal route, caused a significant inhibition of carrageenan-induced rat paw edema with inhibition of 49 ± 13% and 64 ± 9%, respectively.	[61]
*C. multijuga* Hayne	oleoresin extracts	Anti-inflammatory: The hexane, chloroform and methanol fractions obtained from *C. multijuga* oleoresin, given by the oral gavage, caused a significant inhibition of carrageenan-induced rat paw edema.	[100]
*C. multijuga* Hayne	oleoresin extracts	Antinociceptive: The hexane, chloroform and methanol fractions obtained from *C. multijuga* oleoresin, given by the oral gavage, caused a significant inhibition (in a concentration-dependent way) the number of contortions induced by acetic acid and the second phase of formalin-induced licking response. Similar results were observed in the tail flick model; administration of naloxone inhibited the antinociceptive effect of the fractions indicating that copaiba resin maybe acting on opioid receptors.	[100]
*C. multijuga* Hayne	3β-alepterolic acid	Antiproliferative: In vitro cytotoxicity, 20 μM concentration, on AGP01 (human gastric cancer, 8.5% growth inhibition) and SF-295 (human glioblastoma, 21% growth inhibition) cells.	[74]
*C. multijuga* Hayne	3β-alepterolic acid acetate	Antiproliferative: In vitro cytotoxicity, 20 μM concentration, on AGP01 (human gastric cancer, 13% growth inhibition) and SF-295 (human glioblastoma, 18% growth inhibition) cells.	[74]
*C. officinalis* (Jacq.) L.	oleoresin	Antiproliferative: In vivo Walker 256 carcinoma inoculated into the vagina and uterine cervix of female rats, *C. officinalis* oleoresin stimulated tumor growth by 70%.	[101]
*C. officinalis* (Jacq.) L.	oleoresin EO	Anti-inflammatory: Dias and coworkers investigated the immunomodulatory effects of *C. officinalis* oleoresin essential oil on inflammatory cytokines (NO, H_2_O_2_, TNF-α, IFN-γ, and IL-17) in a murine model of experimental autoimmune encephalomyelitis. At a concentration of 100 μg/mL, *C. officinalis* oil inhibited the in vitro production of the inflammatory cytokines, modulating the immune response.	[62]
*C. officinalis* (Jacq.) L.	oleoresin	Antileishmanial: *L. amazonensis* promastigotes (IC_50_ = 20.0 μg/mL).	[48]
*C. officinalis* (Jacq.) L.	oleoresin	Antibacterial: *S. aureus* (MIC = 62.5 μg/mL), MRSA (MIC = 125 μg/mL), *B. subtilis* (MIC = 31.3 μg/mL), *S. epidermidis* (MIC = 31.3 μg/mL), *Enterococcus faecalis* (MIC = 31.3 μg/mL)	[83]
*C. officinalis* (Jacq.) L.	oleoresin	Antibacterial: *Streptococcus mutans* (MIC = 780 μg/mL)	[102]
*C. officinalis* (Jacq.) L.	oleoresin	Antibacterial: *Staphylococcus aureus* (MIC = 312.5 μg/mL)	[103]
*C. officinalis* (Jacq.) L.	agathic acid	Antileishmanial: *L. amazonensis* promastigotes (IC_50_ = 28.0 μg/mL), amastigotes (IC_50_ = 17.0 μg/mL)	[104]
*C. officinalis* (Jacq.) L.	alepterolic acid (=hydroxycopalic acid)	Antileishmanial: *L. amazonensis* promastigotes (IC_50_ = 2.5 μg/mL), amastigotes (IC_50_ = 18.0 μg/mL)	[104]
*C. officinalis* (Jacq.) L.	kaurenoic acid	Antileishmanial: *L. amazonensis* promastigotes (IC_50_ = 28.0 μg/mL), amastigotes (IC_50_ = 3.5 μg/mL)	[104]
*C. officinalis* (Jacq.) L.	methyl copalate	Antileishmanial: *L. amazonensis* promastigotes (IC_50_ = 6.0 μg/mL), amastigotes (IC_50_ = 14.0 μg/mL)	[104]
*C. officinalis* (Jacq.) L.	pinifolic acid	Antileishmanial: *L. amazonensis* promastigotes (IC_50_ = 70.0 μg/mL), amastigotes (IC_50_ = 4.0 μg/mL)	[104]
*C. officinalis* (Jacq.) L.	*ent*-polyalthic acid	Antileishmanial: *L. amazonensis* promastigotes (IC_50_ = 35.0 μg/mL), amastigotes (IC_50_ = 15.0 μg/mL)	[104]
*C. paupera* (Herzog) Dwyer	oleoresin	Antileishmanial: *L. amazonensis* promastigotes (IC_50_ = 11.0 μg/mL).	[48]
*C. paupera* (Herzog) Dwyer	oleoresin	Antibacterial: *B. subtilis* (MIC = 62.5 μg/mL), *Enterococcus faecalis* (MIC = 62.5 μg/mL)	[83]
*C. paupera* (Herzog) Dwyer	copalic acid	Antibacterial: *Bacillus subtilis* (MIC = 3.1–6.3 μg/mL), *Staphylococcus aureus* (MIC = 8–10 μg/mL), *Staphylococcus epidermidis* (MIC = 4–5 μg/mL).	[75]
*C. paupera* (Herzog) Dwyer	*ent*-polyalthic acid	Antibacterial: *Bacillus subtilis* (MIC = 20–30 μg/mL), *Staphylococcus aureus* (MIC = 40–50 μg/mL), *Staphylococcus epidermidis* (MIC = 40 μg/mL).	[75]
*C. paupera* (Herzog) Dwyer	kaurenoic acid	Antibacterial: *Bacillus subtilis* (MIC = 2.5–5 μg/mL), *Staphylococcus aureus* (MIC = 6–8 μg/mL), *Staphylococcus epidermidis* (MIC = 4–6 μg/mL).	[75]
*C. paupera* (Herzog) Dwyer	methyl copalate	Antiproliferative: In vitro cytotoxicity, P-388 (murine lymphoma, IC_50_ = 2.5 μg/mL), A-549 (human lung carcinoma, IC_50_ = 5 μg/mL), HT-29 (human colon carcinoma, IC_50_ = 5 μg/mL), MEL-28 (human melanoma, IC_50_ = 10 μg/mL).	[75]
*C. reticulata* Ducke	oleoresin	Antibacterial: *Porphyromonas gingivalis* (MIC = 6.25 μg/mL), *Streptococcus* spp. (MIC 25–50 μg/mL)	[68]
*C. reticulata* Ducke	oleoresin	Antiproliferative: In vitro cytotoxicity, GM07492-A (human lung fibroblast cells, IC_50_ = 51.85 μg/mL)	[68]
*C. reticulata* Ducke	oleoresin	Anxiolytic: elevated plus-maze test with rats: Oral doses of 100, 400, and 800 mg/kg produced a dose-dependent anxiolytic-like effect over the dose range tested.	[105]
*C. reticulata* Ducke	oleoresin	Insecticidal: Mosquito larvicidal activity (*Culex quinquefasciatus*, LC_50_ = 0.4, 0.9, 39, and 90 μg/mL against the 1st, 2nd, 3rd, and 4th larval instars, respectively)	[106]
*C. reticulata* Ducke	oleoresin	Insecticidal: Mosquito larvicidal activity (*Aedes aegypti*, LC_50_ = 8.9 μg/mL against the 3rd larval instar)	[107]
*C. reticulata* Ducke	oleoresin	Neuroprotective: Rat model of motor cortex excitotoxic injury, *C. reticulata* resin treatment induces neuroprotection by modulating inflammatory response following an acute damage to the central nervous system.	[67]
*C. reticulata* Ducke	oleoresin	Acaricidal: *Rhipicephalus* (*Boophilus*) *microplus* (LC_50_ = 1579 μg/mL)	[108]
*C. reticulata* Ducke	oleoresin	Anti-inflammatory: At a concentration of 500 μg/mL, *C. reticulata* oleoresin oil showed 85% inhibition of NO production in mouse peritoneal macrophages in vitro.	[13]
*C. reticulata* Ducke	oleoresin	Antileishmanial: A β-caryophyllene-rich *C. reticulata* (from Pará state) oleoresin oil showed remarkable activity against *L. amazonensis* promastigotes with IC_50_ of 5.0 μg/mL. Another sample of *C. reticulata* oil (from Acre) with lower concentration of β-caryophyllene was less active (IC_50_ = 22.0 μg/mL).	[48]
*C. reticulata* Ducke	oleoresin EO	Antileishmanial: *C. reticulata* oleoresin essential oil showed significant antileishmanial activity against axenic amastigotes (IC_50_ = 15.0 μg/mL) and intracellular amastigotes (IC_50_ = 20 μg/mL) of *L. amazonensis*. Note that the major component of the oil, β-caryophyllene, was inactive against the amastigotes. Interference with the mitochondrial membrane was suggested as the mechanism for antileishmanial activity.	[109]
*C. reticulata* Ducke	oleoresin	Antinociceptive: Mouse model (acetic acid-induced writhing, tail flick, hot plate), oral administration of *C. reticulata* oil with doses of 30–150 mg/kg significantly showed antinociception, which was reversed with naloxone.	[99]
*C. reticulata* Ducke	oleoresin	Antitrypanosomal: *T. evansi*, in vivo mouse model, doses of 0.63 mL/kg/day over 5 days showed no curative effects. *T. evansi*, in vitro trypomastigotes, 0.5% solution of *C. reticulata* oil showed 100% killing after 6 h.	[86]
*C. reticulata* Ducke	oleoresin EO (Acre)	Antibacterial: *S. aureus* (MIC = 62.5 μg/mL), MRSA (MIC = 125 μg/mL), *B. subtilis* (MIC = 31.3 μg/mL), *S. epidermidis* (MIC = 62.5 μg/mL), *Enterococcus faecalis* (MIC = 62.5 μg/mL)	[83]
*C. reticulata* Ducke	3β-alepterolic acid	Insecticidal: Mosquito larvicidal activity (*Aedes aegypti*, LC_50_ = 87.3 μg/mL against the 3rd larval instar)	[82]
*C. reticulata* Ducke	3β-alepterolic acid acetate	Insecticidal: Mosquito larvicidal activity (*Aedes aegypti*, LC_50_ = 0.8 μg/mL against the 3rd larval instar)	[82]
*Copaifera* spp.	oleoresin	Antibacterial: Oleoresin oils from unidentified species of *Copaifera* showed remarkable antibacterial activity against the Gram-positive *Bacillus subtilis* and *Staphylococcus aureus* (MIC = 5 μg/mL). The oils were inactive against Gram-negative organisms.	[110]
*Copaifera* spp.	agathic acid	Antitrypanosomal: *T. cruzi* epimastigotes (IC_50_ = 86.8 μM), trypomastigotes (IC_50_ = 823 μM), amastigotes (IC_50_ = 14.9 μM)	[111]
*Copaifera* spp.	copalic acid	Antitrypanosomal: *T. cruzi* epimastigotes (IC_50_ = 47.2 μM), trypomastigotes (IC_50_ = 444 μM), amastigotes (IC_50_ = 1.3 μM). Note: β-caryophyllene + copalic acid showed a significant synergistic effect against *T. cruzi* trypomastigotes.	[111]
*Copaifera* spp.	alepterolic acid (=hydroxycopalic acid)	Antitrypanosomal: *T. cruzi* epimastigotes (IC_50_ = 41.2 μM), trypomastigotes (IC_50_ = 453 μM), amastigotes (IC_50_ = 1.8 μM)	[111]
*Copaifera* spp.	kaurenoic acid	Antitrypanosomal: *T. cruzi* epimastigotes (IC_50_ = 167 μM), trypomastigotes (IC_50_ = 596 μM), amastigotes (IC_50_ = 16.5 μM)	[111]
*Copaifera* spp.	methyl copalate	Antitrypanosomal: *T. cruzi* epimastigotes (IC_50_ = 83.3 μM), trypomastigotes (IC_50_ = 377 μM), amastigotes (IC_50_ = 2.5 μM)	[111]
*Copaifera* spp.	pinifolic acid	Antitrypanosomal: *T. cruzi* epimastigotes (IC_50_ = 854 μM), trypomastigotes (IC_50_ = 1630 μM), amastigotes (IC_50_ = 18.6 μM)	[111]
*Copaifera* spp.	*ent*-polyalthic acid	Antitrypanosomal: *T. cruzi* epimastigotes (IC_50_ = 168 μM), trypomastigotes (IC_50_ = 965 μM), amastigotes (IC_50_ = 28.4 μM)	[111]
*Copaifera* spp.	β-caryophyllene	Antileishmanial: *L. amazonensis* amastigotes (IC_50_ = 1.3 μg/mL)	[112]
*Copaifera* spp.	β-caryophyllene	Antitrypanosomal: *T. cruzi* epimastigotes (IC_50_ = 78.4 μM), trypomastigotes (IC_50_ = 1593 μM), amastigotes (IC_50_ = 63.7 μM)	[111]

**Table 4:** MolDock docking energies (kJ/mol) of *Copaifera* diterpenoids with *Leishmania* protein targets.

*Leishmania* Targets	PDB^a^	*E*_dock_ (ave)	*E*_dock_ (min)	Best-Docking Diterpenoid Ligand
Cathepsin B (LdonCatB)	homology	−84.6	−100.6	3α-Alepterolic acid acetate
Cathepsin B (LmajCatB)	homology	−80.8	−93.7	7α-Acetoxyhardwickiic acid
Cyclophilin A (LdonCypA)	3EOV	−83.3	−102.6	*ent*-Pinifolic acid
Deoxyuridine triphosphate nucleotidohydrolase (LmajdUTPase)	2YAY	−85.3	−103.8	19-*O*-Acetyl-1,2-dehydrokautriwaic acid
Dihydroorotate dehydrogenase (LdonDHODH)	3GYE	−89.9	−102.7	7α-Acetoxyhardwickiic acid
Dihydroorotate dehydrogenase (LmajDHODH)	3MHU	−109.2	−126.7	7α-Acetoxyhardwickiic acid
Glucose-6-phosphate isomerase (LmexGPI)	1Q50	−73.0	−85.3	19-*O*-Acetyl-1,2-dehydrokautriwaic acid
Glyceraldehyde-3-phosphate dehydrogenase (LmexGAPDH)	1A7K	−74.2	−83.0	19-*O*-Acetyl-1,2-dehydrokautriwaic acid
Glycerol-3-phosphate dehydrogenase (LmexGPDH)	1N1E	−100.4	−114.3	3α-Alepterolic acid acetate
Methionyl-tRNA synthetase (LmajMetRS)	3KFL	−106.9	−123.0	3α-Alepterolic acid acetate
Nicotinamidase (LinfPnC1)	3R2J	−61.3	−75.9	3β-Alepterolic acid
*N*-Myristoyl transferase (LmajNMT)	4A30	−92.3	−104.0	19-*O*-Acetyl-1,2-dehydrokautriwaic acid
Nucleoside diphosphate kinase b (LmajNDKb)	3NGS	−83.9	−105.8	7α-Acetoxyhardwickiic acid
Nucleoside hydrolase (LmajNH)	1EZR	−80.5	−90.5	7-Acetylbacchotricuneatin D
Oligopeptidase B (LmajOPB)	2XE4	−97.8	−106.1	7α-Acetoxyhardwickiic acid
Phosphodiesterase B1 (LmajPDEB1)	2R8Q	−89.5	−105.8	3β-Alepterolic acid acetate
Phosphomannumutase (LmexPMM)	2I55	−94.2	−117.5	19-*O*-Acetyl-1,2-dehydrokautriwaic acid
Pteridine reductase 1 (LmajPTR1)	1E7W	−93.8	−110.7	Copaiferolic acid
Pyruvate kinase (LmexPYK)	1PKL	−103.4	−113.5	7α-Acetoxyhardwickiic acid
Sterol 14α-demethylase (LinfCYP51)	3L4D	−90.2	−111.3	19-*O*-Acetyl-1,2-dehydrokautriwaic acid
Thiol-dependent reductase I (LinfTDR1)	4AGS	−78.7	−88.8	19-*O*-Acetyl-1,2-dehydrokautriwaic acid
Triosephosphate isomerase (LmexTIM)	2VXN	−90.7	−101.5	3α-Alepterolic acid acetate
Trypanothione reductase (LinfTR)	4APN	−92.5	−109.0	19-*O*-Acetyl-1,2-dehydrokautriwaic acid
Tyrosyl-tRNA synthetase (LmajTyrRS)	3P0J	−92.4	−102.9	Patagonic acid
Uridine diphosphate-glucose pyrophosphorylase (LmajUGPase)	2OEG	−99.9	−113.9	Patagonic acid

^a^ PDB = Protein Data Bank.

**Table 5:** MolDock docking energies (kJ/mol) of *Copaifera* diterpenoids with *Trypanosoma cruzi* protein targets.

*Trypanosoma cruzi* Targets	PDB	*E*_dock_ (ave)	*E*_dock_ (min)	Best-Docking Diterpenoid Ligand
Cruzain	3IUT	−80.2	−92.6	Patagonic acid
Cyclophilin (TcCyp19)	1XQ7	−78.9	−92.0	3β-Alepterolic acid acetate
Deoxyuridine triphosphatase (TcUTPase)	1OGK	−83.4	−101.0	3β-Alepterolic acid acetate
Dihydrofolate reductase—thymidylate synthase (TcDHFR-TS)	3IRN	−93.2	−110.7	7α-Acetoxyhardwickiic acid
Dihydroorotate dehydrogenase (TcDHODH)	3W6Y	−92.5	−109.7	7α-Acetoxyhardwickiic acid
Farnesyl diphosphate synthase (TcFPPS)	3ICZ	−96.2	−109.8	7α-Acetoxyhardwickiic acid
Glyceraldehyde-3-phosphate dehydrogenase (TcGAPDH)	1QXS	−70.3	−85.3	Copaiferolic acid
Hypoxanthine phosphoribosyltransferase (TcHPRT)	1P19	−82.1	−94.4	7α-Hydroxyhardwickiic acid
Nucleoside diphosphate kinase B (TcNDKb)	3PRV	−71.6	−88.4	Crolechinic acid
Old yellow enzyme (=Prostaglandin F2α synthase) (TcPGFS)	3ATY	−85.6	−97.3	Patagonic acid
Pteridine reductase 2 (TcPTR2)	1MXH	−96.8	−118.4	(+)-Hardwickiic acid
Pyruvate kinase (TcPYK)	3QV9	−80.3	−87.4	(−)-Hardwickiic acid
Spermidine synthase (TcSpdSyn)	3BWC	−96.8	−106.8	19-*O*-Acetyl-1,2-dehydrokautriwaic acid
Sterol 14α-demethylase (TcCYP51)	3KLO	−89.5	−101.8	19-*O*-Acetyl-1,2-dehydrokautriwaic acid
Triosephosphate isomerase (TcTIM)	1SUX	−88.2	−100.7	*ent*-Polyalthic acid
Trypanothione reductase (TcTR)	1BZL	−81.9	−95.8	Copaiferolic acid
UDP-galactose mutase (TcUGM)	4DSH	−104.5	−115.7	Copaiferolic acid

**Table 6:** MolDock docking energies (kJ/mol) of *Copaifera* diterpenoids with bacterial protein targets.

Bacterial Protein Targets	PDB	*E*_dock_ (ave)	*E*_dock_ (min)	Best Docking Diterpenoid Ligand
*Pseudomonas aeruginosa* peptide deformylase (PaPDF)	1LRY	−96.3	−113.5	(+)-Hardwickiic acid
*Streptococcus pneumoniae* peptide deformylase (SpPDF)	2AIE	−100.2	−115.4	7α-Acetoxyhardwickiic acid
*Mycobacterium tuberculosis* peptide deformylase (MtPDF)	3E3U	−95.7	−107.3	(+)-Hardwickiic acid
*Escherichia coli* topoisomerase IV (EcTopoIV)	1S16	−100.5	−118.8	7α-Acetoxyhardwickiic acid
*Mycobacterium tuberculosis* DNA gyrase B (MtDNAGyrB)	3ZKD	−101.3	−118.3	3α-Alepterolic acid acetate
*Mycobacterium tuberculosis* protein tyrosine phosphatase (MtPTPB)	2OZ5	−89.2	−107.2	*ent*-Polyalthic acid
*Mycobacterium tuberculosis* UDP-galactopyranose mutase (MtUGM)	4RPL	−92.2	−104.4	19-*O*-Acetyl-1,2-dehydrokautriwaic acid
*Mycobacterium tuberculosis* mycocyclosin synthase (MtCYP121)	5IBE	−87.0	−108.2	19-*O*-Acetyl-1,2-dehydrokautriwaic acid
*Escherichia coli* DNA ligase (EcLigA)	2OWO	−97.8	−108.5	19-*O*-Acetyl-1,2-dehydrokautriwaic acid
*Mycobacterium tuberculosis* DNA ligase (MtLigA)	1ZAU	−89.0	−107.2	19-*O*-Acetyl-1,2-dehydrokautriwaic acid
*Staphylococcus aureus* DNA ligase (SaLigA)	4CC6	−85.2	−97.8	Methyl copalate
*Streptococcus pneumoniae* DNA ligase (SpLigA)	4GLW	−88.1	−109.5	Cativic acid

**Table 7:** MolDock docking energies (kJ/mol) of *Copaifera* diterpenoids with cancer-relevant protein targets.

Cancer-Relevant Protein Targets	PDB	*E*_dock_ (ave)	*E*_dock_ (min)	Best Docking Ligand
Human androgen receptor (HsAR)	5VO4	−67.6	−103.2	Cativic acid
Human aromatase (HsCYP19A1)	5JKW	−97.1	−112.7	*ent*-Pinifolic acid
Human casein kinase II (HsCK2)	5N9K	−83.3	−101.0	3α-Alepterolic acid acetate
Human cyclin-dependent kinase 2 (HsCDK2)	5JQ8	−91.1	−104.1	7α-Acetoxyhardwickiic acid
Human cyclin-dependent kinase 4 (HsCDK4)	2W96	−95.2	−117.6	3α-Alepterolic acid acetate
Human cyclin-dependent kinase 6 (HsCDK6)	5L2S	−87.3	−99.0	(+)-Hardwickiic acid
Murine cyclooxygenase 2 (MmCOX-2)	6COX	−93.7	−106.9	Kolavenic acid
Human DNA (cytosine-5)-methyltransferase 1 (HsDNMT1)	3SWR	−102.7	−121.8	Patagonic acid
Human DNA (cytosine-5)-methyltransferase 3A (HsDNMT3A)	2QRV	−94.3	−113.4	7α-Hydroxyhardwickiic acid
Human epidermal growth factor receptor (HsEGFR)	1XKK	−81.2	−98.8	19-*O*-Acetyl-1,2-dehydrokautriwaic acid
Human estrogen receptor α (HsERα)	1X7E	−96.5	−107.5	19-*O*-Acetyl-1,2-dehydrokautriwaic acid
Human estrogen receptor β (HsERβ)	1U3S	−99.6	−120.1	19-*O*-Acetyl-1,2-dehydrokautriwaic acid
Human heat shock protein HSP 90-α	5J2X	−83.8	−92.6	Cativic acid
Human insulin-like growth factor 1 receptor (HsIGF1R)	3LW0	−86.1	−94.2	Copaiferolic acid
Human 5-lipoxygenase (Hs5-LOX)	3V99	−89.9	−106.2	3α-Alepterolic acid acetate
Human mitogen-activated protein kinase kinase 1 (HsMEK1)	3OS3	−101.5	−113.5	3β-Alepterolic acid acetate
Murine nuclear factor κ-light-chain-enhancer of activated B cells (MmNF-κB)	1VKX	−74.2	−87.7	3α-Alepterolic acid acetate
Human p90 ribosomal protein S6 kinase (HsRSK2) *C*-terminal domain	4D9U	−75.3	−90.7	3β-Alepterolic acid acetate
Human p90 ribosomal protein S6 kinase (HsRSK2) *N*-terminal domain	4NW6	−86.4	−99.7	7α-Acetoxyhardwickiic acid
Murine P-glycoprotein	3G60	−97.6	−116.1	Patagonic acid
Human phosphatidylinositol-4,5-bisphosphate 3-kinase (HsPI3K)	2A5U	−84.5	−97.6	7α-Acetoxyhardwickiic acid
Human topoisomerase I (HsTOPO-I)	1NH3	−83.7	−99.6	Copaiferolic acid
Human topoisomerase IIα (HsTOPO-IIα)	4FM9	−94.2	−105.2	19-*O*-Acetyl-1,2-dehydrokautriwaic acid
Human topoisomerase IIβ (HsTOPO-IIβ)	4J3N	−85.6	−98.9	3α-Alepterolic acid acetate
Bovine tubulin (colchicine binding site)	1SA1	−94.2	−103.8	3α-Alepterolic acid acetate
Bovine tubulin (paclitaxel binding site)	1JFF	−79.1	−88.6	7α-Acetoxyhardwickiic acid
Bovine tubulin (vinblastine binding site)	1Z2B	−89.1	−101.0	(+)-Hardwickiic acid
Human vascular endothelial growth factor receptor (HsVEGFR)	4ASE	−92.9	−105.5	7-acetylbacchotricuneatin D

**Table 8:** MolDock docking energies (kJ/mol) of *Copaifera* diterpenoids with cancer-relevant protein targets.

Inflammation-Relevant Protein Targets	PDB	*E*_dock_ (ave)	*E*_dock_ (min)	Best Docking Ligand
Murine soluble epoxide hydrolase (MmEPHX2)	1CR6	−108.3	−125.6	19-*O*-Acetyl-1,2-dehydrokautriwaic acid
Human soluble epoxide hydrolase (HsEPHX2)	4HAI	−95.1	−104.5	*ent*-Agathic_acid
Human fibroblast collagenase (HsMMP-1)	1CGL	−96.5	−109.0	19-*O*-Acetyl-1,2-dehydrokautriwaic acid
Porcine phospholipase A2 (SsPLA2)	2B03	−100.0	−112.4	(+)-Hardwickiic acid
Human phospholipase A2 (HsPLA2)	1J1A	−95.4	−109.5	7α-Acetoxyhardwickiic acid
Human 5-lipoxygenase (Hs5-LOX)	3V99	−89.9	−106.2	3α-Alepterolic acid acetate
Murine inducible nitric oxide synthase (MmiNOS)	1M8D	−87.5	−110.4	Copaiferolic acid
Human phosphatidylinositol-4,5-bisphosphate 3-kinase γ (HsPI3Kγ)	2A5U	−84.5	−97.6	7α-Acetoxyhardwickiic acid
Human interleukin-1 receptor-associated kinase 4 (HsIRAK4)	5T1S	−90.7	−100.6	7α-Acetoxyhardwickiic acid
Human glutathione *S*-transferase ω-1 (HsGSTO1)	5V3Q	−82.7	−94.2	19-*O*-Acetyl-1,2-dehydrokautriwaic acid
Ovine cyclooxygenase-1 (OaCOX-1)	3N8Z	−62.1	−92.0	Crolechinic acid
Murine cyclooxygenase-2 (MmCOX-2)	6COX	−93.7	−106.9	Kolavenic acid
Human c-Jun *N*-terminal kinase (HsJNK)	4Y46	−83.7	−97.7	19-*O*-Acetyl-1,2-dehydrokautriwaic acid
*Xenopus laevis* inhibitor of κB kinase β (XlIKKB)	3RZF	−88.1	−100.9	Patagonic acid
Human NF-κB essential modulator (HsNEMO)	3BRT	−85.9	−105.3	3α-Alepterolic acid acetate
Human lipid binding protein MD-2 (HsMD-2)	2E59	−71.2	−84.3	19-*O*-Acetyl-1,2-dehydrokautriwaic acid
Human myeloperoxidase (HsMPO)	4C1M	−84.3	−98.8	(+)-Hardwickiic acid
Murine nuclear factor κ-light-chain-enhancer of activated B cells (MmNF-κB)	3DO7	−72.7	−85.9	(+)-Hardwickiic acid
Human p38 mitogen-activated protein kinase (Hsp38MAPK)	1OZ1	−91.3	−116.4	19-*O*-Acetyl-1,2-dehydrokautriwaic acid
Human peroxisome proliferator-activated receptor γ (HsPPARγ)	3ADV	−91.9	−107.6	Copaiferolic acid
Human cAMP-specific 3′,5′-cyclic phosphodiesterase 4D (HsPDE4D)	5K32	−90.2	−107.3	7α-Acetoxyhardwickiic acid
